# The peripheral perfusion index and transcutaneous oxygen challenge test are predictive of mortality in septic patients after resuscitation

**DOI:** 10.1186/cc12788

**Published:** 2013-06-20

**Authors:** Huai-wu He, Da-wei Liu, Yun Long, Xiao-ting Wang

**Affiliations:** 1 Department of Critical Care Medicine, Peking Union Medical College Hospital, Peking Union Medical College, Chinese Academy of Medical Science, 1 Shuaifuyuan, Dongcheng District, Beijing, 100730,China

**Keywords:** Peripheral perfusion index (PI), transcutaneous oxygen/carbon dioxide tensions (PtcO_2_/PtcCO_2_), oxygen challenge test (OCT), septic shock, prognosis

## Abstract

**Introduction:**

The peripheral perfusion index (PI) is a noninvasive numerical value of peripheral perfusion, and the transcutaneous oxygen challenge test (OCT) is defined as the degree of transcutaneous partial pressure of oxygen (PtcO_2_) response to 1.0 FiO_2. _The value of noninvasive monitoring peripheral perfusion to predict outcome remains to be established in septic patients after resuscitation. Moreover, the prognostic value of PI has not been investigated in septic patients.

**Methods:**

Forty-six septic patients, who were receiving PiCCO-Plus cardiac output monitoring, were included in the study group. Twenty stable postoperative patients were studied as a control group. All the patients inspired 1.0 of FiO_2 _for 10 minutes during the OCT. Global hemodynamic variables, traditional metabolic variables, PI and OCT related-variables were measured simultaneously at 24 hours after PiCCO catheter insertion. We obtained the 10min-OCT ((PtcO_2 _after 10 minutes on inspired 1.0 oxygen) - (baseline PtcO_2_)), and the oxygen challenge index ((10min-OCT)/(PaO_2 _on inspired 1.0 oxygen - baseline PaO_2_)) during the OCT.

**Results:**

The PI was significantly correlated with baseline PtcO_2_, 10min-OCT and oxygen challenge index (OCI) in all the patients. The control group had a higher baseline PtcO_2_, 10min-OCT and PI than the septic shock group. In the sepsis group, the macro hemodynamic parameters and ScvO_2 _showed no differences between survivors and nonsurvivors. The nonsurvivors had a significantly lower PI, 10min-OCT and OCI, and higher arterial lactate level. The PI, 10min-OCT and OCI predicted the ICU mortality with an accuracy that was similar to arterial lactate level. A PI <0.2 and a 10min-OCT <66mmHg were related to poor outcome after resuscitation.

**Conclusions:**

The PI and OCT are predictive of mortality for septic patients after resuscitation. Further investigations are required to determine whether the correction of an impaired level of peripheral perfusion may improve the outcome of septic shock patients.

## Introduction

Blood flow is diverted from the peripheral tissue to vital organs during circulation shock. It is assumed that peripheral tissue is the first tissue bed to sacrifice in shock and the last to be reperfused in resuscitation [1,2]. Tissue perfusion is commonly evaluated indirectly by subjective symptoms and imprecise signs, but they are not direct quantitative measures of peripheral tissue perfusion. With the development of new techniques, quantitatively assessment of peripheral tissue perfusion has become popular in clinical practice. The peripheral perfusion index (PI) is derived from the photoelectric plesthysmographic signal of pulse oximetry and has been shown to reflect changes in peripheral perfusion [3,4]. A PI value of 1.4 indicates the presence of poor peripheral perfusion in critically ill patients [3]. Transcutaneous oxygen/carbon dioxide tensions (PtcO_2_/PtcCO_2_) monitoring can provide continuous displays of data, and have been used in infants and premature neonates as a surrogate measure of arterial blood gases [5]. Because thicker epidermal layers cause more discrepancy between PtcO_2 _and arterial pressure of oxygen (PaO_2_), PtcO_2 _and PtcCO_2 _have been used to represent tissue oxygenation and perfusion in critically ill adult patients [6-8]. In addition, the oxygen challenge test (OCT) is another new method to reflect cellular O_2 _deficit. Recent reports using the noninvasive PtcO_2 _have observed a relationship between low oxygen challenge test values to mortality and new organ failure [9,10].

However, septic shock, in contrast to other forms of shock associated with peripheral vasodilation, is known as 'warm shock'. During septic shock resuscitation, perfusion assessment is difficult and usually a complex determination. The value of peripheral tissue perfusion is controversial in systemic sepsis [11]. To the best of our knowledge, no one has quantified PI to outcome in patients with septic shock. The noninvasive peripheral perfusion potential value to predict outcome remains to be established in septic patients. This study was conducted as a prospective evaluation of the predictive value of the noninvasive peripheral perfusion indicators, traditional metabolic variables, and global hemodynamic parameters in septic patients after resuscitation. This could provide valuable insights about perfusion monitoring in septic patients and help to delineate the potential role and limitations of noninvasive peripheral perfusion as a target for resuscitation.

This study has been partially presented in abstract form [12].

## Patients and methods

The Institutional Research and Ethics Committee of the Peking Union Medical College Hospital approved this study for human subjects. Informed consent was obtained from all patients or next-of-kin before data were included into the study.

When the research team was available, all adult patients within 48 h after the onset of severe sepsis or septic shock sequentially admitted to the Department of Critical Care Medicine of Peking Union Medical College Hospital, who required PiCCO-Plus cardiac output monitoring and mechanical ventilation for resuscitation, were eligible for the study. The attending intensivists decided PiCCO catheter placement according the severity of the patient's condition after the early hemodynamic support. The local reference standard of PiCCO catheter insert is: (1) tissue perfusion or organ function has not improved after early goal-directed therapy [13]; (2) according to the patient's clinical condition, it is difficult for the attending doctor to make a clinical decision of infusion fluid or pressor. Patients excluded from the study were as follows: fraction of inspired oxygen concentration (FiO_2_) >80%; were pregnant; aged <18 years; were not expected to live; were brain dead; due to the decision to withhold or withdraw treatments. Septic shock was defined as severe sepsis with sepsis-induced hypotension persisting, despite adequate fluid resuscitation, and requiring the administration of vasopressors [14].

Twenty stable patients during the first 24 h of the Department of Critical Care Medicine admission who were ventilated and sedated, without infection, and admitted for postoperative monitoring were measured and considered to be the control group.

### Patient management

All the patients needed mechanical ventilation and sedatives. All septic shock patients received broad-spectrum antibiotic coverage. The local hemodynamic support algorithm of septic shock patients: the early goal-directed therapy was as follows: central venous pressure was 8 to 12 mmHg; mean arterial pressure was above 65 mmHg; urine output was above 0.5 ml/kg of body weight (except in the patients with acute renal failure), and the central venous oxygen saturation (ScvO_2_) was at 70% or more [13]. The advanced goal of hemodynamic support (after PiCCO catheter placement) was as follows: fluid loading was guided according to pulse pressure variation in ventilated patients (pulse pressure variations below 13% or lack of response to passive leg raising or no respiratory variations of the inferior vena cava diameter (Cx Cart; Phillips Ultrasound, Bothell, WA, USA)); systemic vascular resistance index >1000 dyne/sec/cm^5^; after the macro hemodynamic variables were optimized, if the lactate was not decreasing, sources for the elevation of lactate were sought, and/or maneuvers to lower oxygen consumption were instituted (fever control, increase in sedation/pain medication). The intensivists were blinded for the results of the peripheral perfusion variables.

### Measurements

Information collected at enrollment included demographic characteristics such as age; sex; Acute Physiology and Chronic Health Evaluation II score (APACHE II) [15]; Sequential Organ Failure Assessment (SOFA) score [16]; and primary site and type of infection. The global hemodynamic, traditional metabolic, and PI and OCT variables were measured simultaneously at 24 h after PiCCO catheter insertion.

Simultaneous basic blood gases from the arterial, central venous catheters were obtained (the placement of a central venous catheter in the superior vena cava was confirmed by chest radiography). Blood gas samples were taken anerobically in 3 ml heparinized syringes (PL67BP; BD Diagnostics, Plymouth, UK) and analyzed on blood gas bedside machines (GEM Premier 3000, model 5700; Lexington, MA, USA) or (ABL90: Radiometer, Copenhagen, Denmark). The thermodilution cardiac output was measured by injecting 15 ml of 0.9% saline at 0°C for the PiCCO-Plus (the PiCCO system: Pulsion Medical System, Munich, Germany). Three cardiac outputs, which were within 10% of each other, were obtained and averaged. These global hemodynamic variables were recorded simultaneously.

Transcutaneous sensors (TCM4 Transcutaneous Oxygen/Carbon Dioxide Monitoring System; Radiometer, Copenhagen, Denmark) having previous validation studies were calibrated to known oxygen and carbon dioxide levels according to the manufacturer's guidelines. The sensors were attached to the patients' upper torso below the clavicle in nonbony areas, and skin was prepared with alcohol and shaved when necessary. Areas of injury such as hematomas were avoided. With a total of 15 min equilibration time, heating of the probe to 44°C required 5 min. Baseline PtcO_2 _and PtcCO_2 _were recorded. The OCTs were performed (FiO_2 _was increased to 1.0 for 10 min), and the new PtcO_2 _value was recorded every 1 min. The values for PaO_2 _were examined on inspired of 1.0 FiO_2_. PI and pulse oxygen saturation percentage (SpO_2_) were measured in the index finger by using the IntelliVue MP70 monitor (Philips Medical Systems, Boblingen, Germany). The MP70 system calculates the PI as the ratio between the pulsatile component and the nonpulsatile component of the light reaching the light-sensitive cell of the pulse oximetry probe. The ambient temperature of the room was kept constant at approximately 23 to 25°C (climate controlled) during all phases of testing.

### Study definitions

The PtcO_2 _index is baseline PtcO_2_/baseline PaO_2_; 10min-OCT is (PtcO_2 _after 10 minutes on inspired 1.0 oxygen) - (baseline PtcO_2_); the OCI is (10min-OCT)/((PaO_2 _on inspired 1.0 oxygen) - (baseline PaO_2_)); survival is leaving the ICU alive for that admission.

### Statistical analysis

A descriptive analysis was performed. All data were expressed as mean ± standard deviation, unless otherwise specified. A Mann-Whitney test was used to compare groups on continuous variables, and chi-square and Fisher's exact tests were used to compare categorical variables. Comparisons of two continuous variables were performed using a linear regression. Discrimination of values was assessed with the receiver operating characteristic analysis. All comparisons were two-tailed, and *P *<0.05 was required to exclude the null hypothesis. The areas under the ROC curves were compared using a Hanley-McNeil test [17]. The statistical analysis was performed by using the software package SPSS version 13.0 (SPSS Inc. Chicago, IL, USA), and MedCalc 11.4.3.0 software (MedCalc, Mariakerke, Belgium).

## Results

From July 2009 to May 2012, a total of 66 patients were enrolled. Fifty-five septic shock patients were screened for the study, and nine septic shock patients were not included in the analysis because of the failure of the correction of ScvO_2 _(ScvO_2 _<60%). Twenty-six of the 46 septic shock patients were also engaged in a previous reported study [18]. The median time between the start of the PiCCO catheter insertion and admission to the ICU was 16 h in the study group. The ICU mortality in the 46 septic shock patients was 43% (20/46). In this population, there were no differences in the using of norepinephrine (NE), the APACHE and SOFA score and white blood cell (WBC) count between the survivors and the nonsurvivors. The characteristics of the primary infection sites were as follows: 24/46 (52%) cases of lung; 10/46 (22%) cases of abdomen; 1/46 (2%) cases of urinary tract; 2/46 (4%) cases of soft tissue; 1/46 (2%) cases of bloodstream; 8/46 (17%) cases of an unknown source. Characteristics of the septic shock patients are shown in Table 1. The control group included 20 hemodynamically stable patients without sepsis admitted for postoperative monitoring. The characteristics of surgery sites were as follows: 16/20 (80%) cases of abdomen, 4/20 (20%) cases of other sites. None of these patients was treated with pressor agents. Demographics and clinical characteristics of the study groups and the control groups are shown in Table 2; the mean arterial pressure (MAP) had been corrected in all the septic shock patients at the measurement stage.

**Table 1:** Characteristics of the septic patients (*n *= 46).

Variables	Survivor *n *= 26	Nonsurvivor *n *= 20	*P *value
Age (years)	58 ± 16	67 ± 16	0.080
Sex (female/male)	8:18	8:12	0.515
APACHE II score	18 ± 6	20 ± 6	0.069
SOFA score	9 ± 2	10 ± 2	0.056
Ramsay score	5 ± 0.8	5.5 ± 0.7	0.583
NE	0.59 ± 0.67	0.74 ± 0.62	0.235
WBC	12.7 ± 7.8	9.1 ± 5.9	0.126

^a^*P *<0.05 for survivor group vs. nonsurvivor group. APACHE, Acute Physiology and Chronic Health Evaluation; SOFA, Sequential Organ Failure Assessment score; NE, norepinephrine (μg/kg/min); WBC, white blood cell count (X 10^9/L).

**Table 2:** Comparison of the septic group and the control group.

Variables	Control group *n *= 20	Septic group *n *= 46	*P *value
Age (years)	59 ± 10	62 ± 16	0.442
Sex (female/male)	9:11	16:30	0.432
APACHE II score	12 ± 4	19 ± 6	0.000^a^
SOFA score Ramsay score	2 ± 2 5.1 ± 0.3	9 ± 2 5 ± 1	0.000^a^ 0.008 ^a^
MAP	93 ± 9	87 ± 12	0.024 ^a^
SpO_2 _(%)	100 ± 1	99 ± 2	0.193
Lactate (mmol/L)	3.2 ± 2.2	3.8 ± 3.9	0.950

^a^*P *<0.05 for control group vs. septic group. APACHE, Acute Physiology and Chronic Health Evaluation; SOFA, Sequential Organ Failure Assessment score; MAP, mean arterial pressure (mmHg); SpO_2_, arterial oxygen saturation measured by pulse oximetry.

### Global, metabolic, and peripheral perfusion variables in different groups

There were no differences in SpO_2_, PtcCO_2_, and arterial lactate level between the control patients and the septic patients (Table 2). The control group had a higher baseline PaO_2_, baseline PtcO_2_, PaO_2 _(on FiO_2 _1.0), MAP, 10min-OCT and PI than the septic group (Table 3). There was no difference in PtcO_2 _index and OCI between the two groups.

**Table 3:** Peripheral perfusion related-variables in the different groups

Variables	Controls n = 20	All sepsis *n *= 46	Survivor sepsis *n *= 26	Nonsurvivor sepsis *n *= 20
PI	2.58 ± 0.68^a,c^	1.37 ± 1.43	1.95 ± 1.5^c^	0.6 ± 0.86
PtcO_2_	124 ± 45^a,b,c^	73 ± 24	79 ± 21	66 ± 26
PaO_2 _(on basic FiO_2_)	179 ± 70^a,b,c^	119 ± 45	125 ± 51	111 ± 37
PtcO_2 _index	0.71 ± 0.29	0.67 ± 0.22	0.69 ± 0.21	0.64 ± 0.24
PtcCO_2_	36 ± 7	36 ± 9	38 ± 7	34 ± 12
PaCO_2_	35 ± 6	36 ± 8	37 ± 6	35 ± 10
10min-OCT	247 ± 65^a,b,c^	145 ± 99	186 ± 75^c^	93 ± 103
PaO_2 _(on 1.0 FiO_2_)	489 ± 106^a,b,c^	343 ± 122	371 ± 116	307 ± 123
OCI	0.83 ± 0.2^c^	0.66 ± 0.35	0.79 ± 0.28^c^	0.49 ± 0.36

^a^*P *<0.05 for controls vs. all sepsis; ^b^*P *<0.05 for controls vs. survivor sepsis; ^c^*P *<0.05 for controls vs. nonsurvivor sepsis. PI, peripheral perfusion index measured by pulse oximetry; FiO_2_, fraction of inspired oxygen concentration; PtcO_2_, transcutaneous partial pressure of oxygen (mmHg); PaO_2_, arterial pressure of oxygen (mmHg); PtcCO_2_, transcutaneous partial pressure of carbon dioxide (mmHg); PaCO_2_, arterial pressure of carbon dioxide (mmHg); 10min-OCT, 10 minute oxygen challenge test value (mmHg); OCI, oxygen challenge index.

Global, metabolic and peripheral perfusion variables after resuscitation of the septic patients according to survival are shown in Table 3 and Table 4. In this study group, the macro hemodynamic-related parameters, baseline PtcO_2_, baseline PtcCO_2_, PaO_2 _(on FiO_2 _1.0), PtcO_2 _index and ScvO_2 _showed no differences between survivors and nonsurvivors. Statistically significant variables between the survivors and the nonsurvivors included arterial lactate level (2.3 ± 2 vs. 5.6 ± 4.9mmol/l, *P *<0.0001), PI (1.95 ± 1.5 vs. 0.6 ± 0.86, *P *<0.0001), 10min-OCT (186 ± 75 vs. 93 ± 103 mmHg, *P *= 0.001) and OCI (0.79 ± 0.28 vs. 0.49 ± 0.36, *P *= 0.01).

**Table 4:** Global and metabolic variables in the septic patients according to their survival

Variables	Total *n *= 46	Survivor *n *= 26	Nonsurvivor *n *= 20	*P *value
Blood temp (°C)	38 ± 1.1	38 ± 0.9	38.1 ± 1.4	0.564
PEEP	7 ± 3	7 ± 3	7 ± 2	0.910
Basic FiO_2_	49 ± 9	47 ± 8	51 ± 9	0.078
MAP	87 ± 12	88 ± 12	85 ± 12	0.513
CVP	11 ± 3	12 ± 3	11 ± 4	0.319
CI	3.7 ± 1	3.9 ± 0.8	3.6 ± 1.3	0.111
SVRI	1705 ± 559	1676 ± 474	1742 ± 664	0.982
GEDVI	805 ± 162	744 ± 125	844 ± 197	0.341
ScvO_2 _(%)	71 ± 8	72 ± 8	69 ± 8	0.156
Pv-a CO_2_	6 ± 3	5 ± 3	7 ± 4	0.144
Lactate (mmol/L)	3.8 ± 3.9	2.3 ± 2	5.6 ± 4.9	0.000^a^

^a^*P *<0.05 for survivor vs. nonsurvivor. PEEP, positive end-expiratory pressure (cmH_2_O); FiO_2_, fraction of inspired oxygen concentration; MAP, mean arterial pressure (mmHg); CVP, central venous pressure (mmHg); CI, cardiac index (L/min/m^2^); SVRI, systemic vascular resistance index (dyne/sec/cm^5^); GEDVI, global end-diastolic volume index (ml/m^2^); ScvO_2_, central venous oxygen saturation; Pv-a CO_2 _,difference between central venous and arterial PCO_2 _(mmHg).

There were 26 patients in septic group with ScvO_2 _≥70% after resuscitation, and the mortality was 9/26 (35%) in this subgroup. Statistically significant variables between the survivors and the nonsurvivors included arterial lactate level (2.3 ± 1.5 vs. 5.0 ± 5.2mmol/l, *P *= 0.034), PI (2.2 ± 1.7 vs. 0.87 ± 1.1, *P *= 0.013).

In the whole group, the PI was not correlated with the baseline PtcCO_2_, PtcO_2 _index and ScvO_2_. In contrast, the PI was correlated with baseline PtcO_2 _(r = 0.298, *P *= 0.015), 10min-OCT (r = 0.573, *P *<0.0001), OCI (r = 0.395, *P *= 0.001), lactate (r = -0.309, *P *= 0.012). There is a significantly positive relationship between PaCO_2 _and PtcCO_2 _(r = 0.874, *P *<0.0001).

### Metabolic and peripheral perfusion variables as predictors of mortality

**Figure 1:**
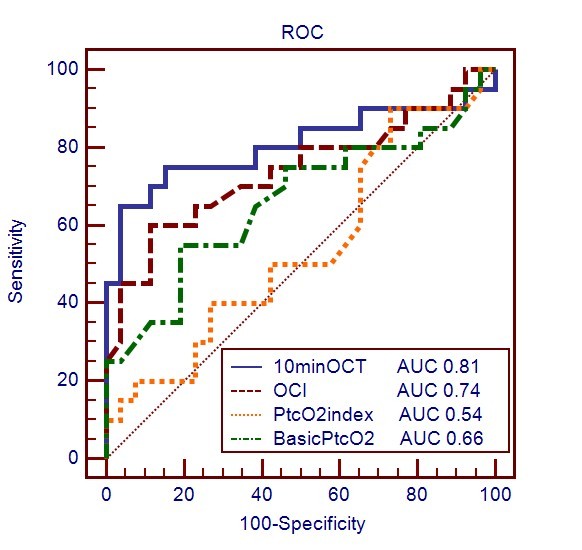
**ROC curves comparing the ability of 10min-OCT, OCI, baseline PtcO_2 _and PtcO_2 _index to discriminate ICU mortality**. 10min-OCT, 10 minute oxygen challenge test value (mmHg); OCI, oxygen challenge index; PtcO_2_, transcutaneous partial pressure of oxygen (mmHg); PtcO_2 _index, transcutaneous partial pressure of oxygen index; AUC, area under the receiver operating characteristic (ROC) curve.

**Figure 2:**
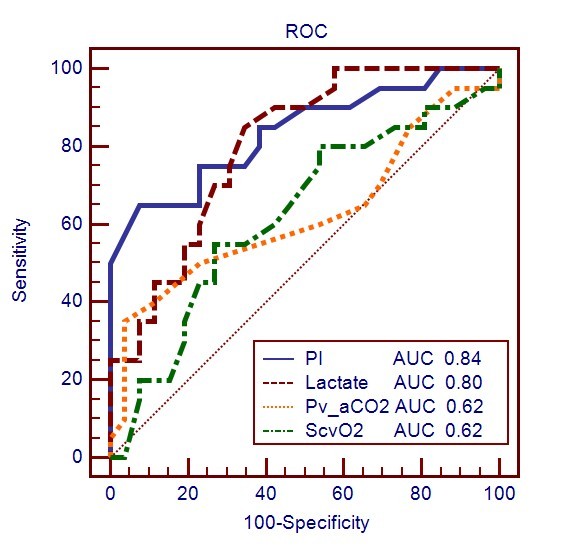
**ROC curves comparing the ability of PI, lactate, Pv-a CO_2 _and ScvO_2 _to discriminate ICU mortality**. PI, peripheral perfusion index measured by pulse oximetry; Pv-aCO_2_, difference between central venous and arterial PCO_2 _(mmHg); ScvO_2_, central venous oxygen saturation; AUC, area under the receiver operating characteristic (ROC) curve.

The cutoff value and areas under the receiver operating characteristic (ROC) curves for the related variables used for predicting ICU mortality are shown in Table 5. The cutoff of the PI value was ≤0.2 for predicting ICU mortality, resulting in a sensitivity of 65% and a specificity of 92.3%. The PI, 10min-OCT and OCI predicted the ICU mortality with an accuracy that was similar to arterial lactate level, and the PI was significantly better than the difference between central venous and arterial PCO_2 _(P(v-a)CO_2_) and ScvO_2_. In these PtcO_2_-related parameters, the 10min-OCT and OCI were significant predictors of lethal outcome, but not baseline PtcO_2 _and PtcO_2 _index. For predicting ICU mortality, a threshold of 10min-OCT at 66 mmHg was associated with a sensitivity of 65% and a specificity of 96.2%, and a cutoff of OCI at 0.55 was associated with a sensitivity of 60% and a specificity of 88.5%. The ROC curves of baseline PtcO_2_, PtcO_2 _index, 10min-OCT and OCI are shown in Figure 1, and the ROC curves of PI, lactate, ScvO_2 _and Pv-aCO_2 _are shown in Figure 2.

**Table 5:** Comparison of the areas under the ROC curves for predicting ICU mortality in the septic patients.

	ROC area	95% CI	Cutoff value	Sensitivity (%)	Specificity (%)
Lactate	0.80	0.658-0.905	4.2	45	84.62
Pv-a CO_2 _	0.62^a^	0.469-0.762	7	40	88.46
PI	0.84	0.698-0.929	0.2	65	92.3
ScvO_2_	0.62^a^	0.468-0.762	0.67	55	73.08
PtcO_2_	0.66^b^	0.508-0.795	61	55	80.8
PtcO_2 _index	0.54^c^	0.387-0.688	0.51	30	76.92
10min-OCT	0.81	0.662-0.907	66	65	96.2
OCI	0.74	0.588-0.857	0.55	60	88.5

^a^*P ***<**0.05 for comparison of Pv-a CO_2_, ScvO_2_vs, PI; ^b^*P *<0.05 for comparison of PtcO_2 _vs. PI, lactate; ^c^*P *<0.05 for comparison of PtcO_2 _index vs. 10min-OCT, OCI, PI and lactate. ROC, receiver operating characteristic; CI confidence interval; Pv-a CO_2 _, difference between central venous and arterial PCO_2 _(mmHg); PI, peripheral perfusion index measured by pulse oximetry; ScvO_2_, central venous oxygen saturation; 10min-OCT, 10 minute oxygen challenge test value (mmHg); OCI, oxygen challenge index.

## Discussion

The main finding of our study was that lower peripheral perfusion and higher metabolic variables are associated with increased ICU mortality in septic patients after resuscitation. The PI, 10min-OCT and OCI predicted the ICU mortality with an accuracy that was similar to arterial lactate level. Thus, peripheral perfusion monitoring appears as a simple but powerful tool to assess global resuscitation status and outcome.

As we know, this is the first study to explore the relationship between PI and ICU mortality in septic patients after resuscitation. We found that the cutoff of the PI value was ≤0.2 for predicting ICU mortality, resulting in a sensitivity of 65% and a specificity of 92.3%. The PI, which was defined as the ratio of the pulsatile to nonpulsatile component of the pulse oximetry plethysmograph, is used as a simple and accurate indication of changes in digital blood flow. It has been suggested as a reliable and early indicator of regional block success, and known to increase due to the effect of autonomic blockade during spinal anesthesia [4,19,20]. Some studies showed that PI could be used too as a predictor of early adverse respiratory neonatal outcome after elective cesarean delivery [21].

The change of the finger PI resulted from the blood volume pulsations, the dispensability of the vascular wall and the intravascular pulse pressure, which can be complicated in critically ill adult patients [22,23]. Therefore, many factors can impact the PI value (temperature, level of consciousness, pain and other stressful stimuli, endogenous catecholamines, vasopressors). All the patients needed mechanical ventilation and sedatives, and there were no difference in temperature, Ramsay score, and the dose of NE between survivors and nonsurvivors in the study group. Therefore, the PI was relatively comparable in this study. Our study found that PI was significantly lower in the patients with septic shock than in the control group. One can argue that the using of NE may have an effect on PI in the septic patients. However, the nonsurvivors also had a significantly lower PI than survivors in the patients with septic shock. The correlation between hyperlactatemia and PI in our patients could be related to the presence of tissue hypoperfusion. We demonstrated that a value of PI below 0.2 was associated with an increased risk of ICU mortality. Recently, van Genderen and colleagues found that persistent peripheral and microcirculatory perfusion alterations after out-of-hospital cardiac arrest are associated with organ failure and death, independent of systemic hemodynamics. They reported the PI value was 0.3 ± 0.28 in the nonsurvivor at 24 h after rewarming, which is similar to our results in the nonsurvivor after resuscitation [24].

The impact of possible therapy aimed at increasing the PI was not explored in current study. There is a need of further investigations to test such hypothesis. In our study, we investigated the relationship between PI and PtcO_2_-related parameters in critically ill patients. The PI was significantly correlated with baseline PtcO_2_, 10min-OCT and OCI. We concluded that all these parameters are dependent on peripheral blood flow, and reflect peripheral tissue perfusion.

PtcO_2_/PtcCO_2 _is the direct measurement of the local partial pressure of oxygen/carbon dioxide, achieved by placing a Clark polarographic oxygen/carbon dioxide electrode on the skin surface. When the circulation is normal, the PtcO_2 _reflects PaO_2_, but in low flow state, it always underestimates PaO_2_. Shoemaker *et al*. attempted the translation of PtcO_2 _values to cardiac hemodynamics, when they explored the dependence of PtcO_2 _on PaO_2 _using a PtcO_2 _index (PtcO_2_/PaO_2_) to assess the severity of low flow shock [6]. A PtcO_2 _index below 0.7 has been associated with hemodynamic failure in adult patients [25,26]. The PtcO_2 _index has been used to estimate the adequacy of cardiac output and peripheral blood flow. The OCT is another method to determine the situation of circulation using the noninvasive PtcO_2 _technique. In nonshock states, PtcO_2 _correlates with PaO_2 _to strongly increase response to the FiO_2_. When peripheral perfusion severely decreases, the PtcO_2 _values will deviate from their relationship with the arterial tensions and lack of response to a FiO_2 _of 1.0. In our previous study, we showed that an OCT could improve the diagnostic accuracy of the PtcO_2 _in estimating the cardiac index (CI) value, and a cutoff 10min-OCT value of 53 mmHg has a good positive predictive value and positive likelihood ratio for predicting low CI value [18].

We mainly explored the correlation between the outcome and the OCT in this study. We found that the control group had a higher baseline PaO_2_, PaO_2 _(on FiO_2 _1.0), 10min-OCT than the septic shock group. Therefore, we speculated the control group had a better lung function. However, there were no difference of baseline PaO_2 _and PaO_2 _(on FiO_2 _1.0) between the survivors and nonsurvivors. Hence, PtcO_2 _showing a poor response of FiO_2 _1.0 in the nonsurvivor was not due to the lung function, but due to a poor peripheral tissue flow. Moreover, the 10min-OCT and OCI as a predictor of lethal outcome were more sensitive and specific than baseline PtcO_2 _and PtcO_2 _index in the patients with septic shock. For predicting ICU mortality, a threshold of 10min-OCT at 66 mmHg was associated with a sensitivity of 65% and a specificity of 96.2%, and a cutoff of the OCI at 0.55 was associated with a sensitivity of 60% and a specificity of 88.5%. In addition, there is another hypothesis for the OCT to determine tissue perfusion. If there is a cellular oxygen deficit, then the additional dissolved oxygen after the oxygen challenge would be utilized, resulting in a minimum rise in PtcO_2_. The OCT as a method to assess tissue perfusion mainly described in adult patients by using intramuscular PO_2, _as in the previous study [27,28]. Yu *et al*. reported an incremental change of less than 21 mmHg in PtcO_2 _in response to a 1.0 FiO_2 _has been associated with mortality reports during 5min-OCT [9]. Our previous study found that there were substantial differences between the oxygen challenge at 5 minutes and 10 minutes, with the PtcO_2 _value at 10 minutes always being higher than at 5 minutes [18]. The ideal response time of PtcO_2 _on OCT is not well established in critically ill adult patients. Shah *et al*. suggested that the ideal time for the OCT is at least 10 minutes in the evaluation of patients with hypoxic wounds [29]. Further studies are necessary to identify which is the ideal time interval for the OCT. In addition, a multitude of factors can impact PtcO_2_, and the regulation of skin microcirculation during increasing normobaric oxygen is complex [30]. Therefore, the application of an OCT to assess cellular oxygen deficit still needs further investigation.

There are several advantages of the PI and OCT in comparison with the current global endpoints of resuscitation: (1) the peripheral perfusion indicators may provide a specific endpoint of resuscitation rather than the average survival values of ScvO_2_; (2) the peripheral perfusion monitors may provide information on internal organ perfusion with the assumption that the peripheral tissue is the last to perfuse during shock resuscitation; (3) the techniques are noninvasive, and the equipment is readily available and inexpensive. Its importance will be affirmed if peripheral perfusion monitoring during shock resuscitation becomes routine, and if the critical values defined in this paper are validated by further study.

In recent years, tissue oxygen saturation (StO_2_) has been applied to estimate regional perfusion by near-infrared spectroscopy (NIRS), and the changes of StO_2 _during a vascular occlusion test (VOT) have been used as a marker of microvascular reactivity [31-35]. Lima showed that PI correlates with StO_2_, and that peripheral circulation influences StO_2 _resting values and the StO_2 _reoxygenation rate [36,37]. In addition, the changes of PI and PtcO_2 _could be used to evaluate microvascular reactive hyperemia as during a VOT [38,39]. Therefore, we infer that all three (StO_2_, PI, and PtcO_2_) indicators are used to assess regional perfusion and reactive hyperemia with close values.

### Limitations

Several limitations should be acknowledged. First, the study period may be considered not long enough to evaluate other relevant clinical outcomes and the selected time points are relatively arbitrary. A small number of subjects at a single time point is an important issue and our findings should be confirmed in a larger multicenter study before being translated into regular clinical practice. Second, one can argue that the assessment of these objective parameters requires stability of a number of factors that might affect tissue perfusion and oxygenation. To reduce those uncertainty factors, we strictly stabilized the related parameters (ambient temperature, level of consciousness, hemodynamic status). Third was our failure to directly determine skin microcirculation, local blood flow and subjective peripheral tissue perfusion indicators in this study. Fourth, one can argue that this chest skin circulation is not representative of other site circulations. Recently, some studies have been done to explore the value of evaluating peripheral tissue perfusion of other sites in septic shock patients, such as thenar, deltoid, masseter, and ear sites [40,41]. Peripheral tissue perfusion monitoring from different parts may have different values. According to previously published studies on critically ill patients [8-10], we chose the chest site as a relatively standard location for PtcO_2 _monitoring. Moreover, the PI could be considered as a method to assess the finger tip circulation in this study. We found that the two site-related parameters were both predictive for outcome, and there was a significant relationship between chest and finger tip site-related parameters. Further studies should be done to test the clinical worth of PtcO_2 _in other sites in critically ill patients. Fifth, MAP had been corrected in all the septic shock patients at the measurement stage, but not for ScvO_2_. It is difficult for an observational study to guarantee the ScvO_2 _≥70% in clinical practice after resuscitation. In our study, 57% of the septic patients' ScvO_2 _were equal or greater than 70% after resuscitation. We excluded nine septic patients in the analysis with low ScvO_2 _(<60%) after resuscitation, and these patients had high lactate and poor peripheral perfusion. We acknowledge that the using of ScvO_2 _<60% to reveal the imbalance between oxygen delivery and oxygen consumption is relatively arbitrary. According to early goal-directed therapy, ScvO_2 _≥70% is used to reflect the balance between oxygen delivery and oxygen consumption. Therefore, we analyzed a subgroup of septic shock patients with ScvO_2 _≥70%, and we found these peripheral indicators still work. This revealed that peripheral perfusion parameters may provide a specific endpoint of resuscitation after ScvO_2 _≥70%.

## Conclusions

The PI and OCT are predictive of mortality for septic patients after resuscitation, comparable to arterial blood lactate. Further investigations are required to determine whether the correction of an impaired peripheral tissue value may improve the outcome of these patients.

## Key messages

• The PI and OCT are predictive of mortality for septic patients after resuscitation, comparable to arterial blood lactate.

• For predicting ICU mortality, the cutoff of the PI value was ≤0.2 resulting in a sensitivity of 65% and a specificity of 92.3%, and a threshold of 10min-OCT at 66 mmHg was associated with a sensitivity of 65% and a specificity of 96.2%.

• The peripheral perfusion indicators may provide a specific endpoint of resuscitation.

## Abbreviations

APACHE: Acute Physiology and Chronic Health Evaluation; CI: cardiac index; CVP: central venous pressure; FiO_2: _fraction of inspired oxygen concentration; GEDVI: global-heart end-diastolic volume index; ICU: intensive care unit; MAP: mean arterial pressure; NE: norepinephrine; NIRS: near-infrared spectroscopy; OCI: oxygen challenge index; OCT: oxygen challenge test; PaCO_2_: arterial pressure of carbon dioxide; PaO_2_: arterial partial pressure of oxygen; PEEP: positive end-expiratory pressure; PI: peripheral perfusion index; PtcCO_2_: transcutaneous partial pressure of carbon dioxide; PtcO_2_: transcutaneous partial pressure of oxygen; Pv-aCO_2_: difference between central venous and arterial PCO_2_; ROC: receiver operating characteristic; ScvO_2_: central venous oxygen saturation; SpO_2_: pulse oxygen saturation percentage; SOFA: Sequential Organ Failure Assessment; StO_2_: tissue oxygen saturation; SVRI: systemic vascular resistance index; VOT: vascular occlusion test; WBC: white blood cell count; 10min-OCT: 10 minute oxygen challenge test value.

## Competing interests

The authors declare that they have no competing interests.

## Authors' contributions

DL conceived and designed the study, interpreted data and helped draft the manuscript. HWH participated in the study conception and design, recruited patients, collected data, performed the statistical analysis, interpreted the data and drafted the manuscript. YL and XTW participated in patient recruitment, data collection, technical support and contributed to the critical review of the manuscript. All of the authors read and approved the final manuscript.
